# Understanding Counterfactuality: A Review of Experimental Evidence for the Dual Meaning of Counterfactuals

**DOI:** 10.1111/lnc3.12175

**Published:** 2016-02-03

**Authors:** Eugenia Kulakova, Mante S. Nieuwland

**Affiliations:** ^1^ Centre for Cognitive Neuroscience, Department of PsychologyUniversity of Salzburg; ^2^ Department of Psychology, School of Philosophy, Psychology and Language SciencesUniversity of Edinburgh

## Abstract

Cognitive and linguistic theories of counterfactual language comprehension assume that counterfactuals convey a dual meaning. Subjunctive‐counterfactual conditionals (e.g., ‘If Tom had studied hard, he would have passed the test’) express a supposition while implying the factual state of affairs (*Tom has not studied hard and failed*). The question of how counterfactual dual meaning plays out during language processing is currently gaining interest in psycholinguistics. Whereas numerous studies using offline measures of language processing consistently support counterfactual dual meaning, evidence coming from online studies is less conclusive. Here, we review the available studies that examine online counterfactual language comprehension through behavioural measurement (self‐paced reading times, eye‐tracking) and neuroimaging (electroencephalography, functional magnetic resonance imaging). While we argue that these studies do not offer direct evidence for the online computation of counterfactual dual meaning, they provide valuable information about the way counterfactual meaning unfolds in time and influences successive information processing. Further advances in research on counterfactual comprehension require more specific predictions about how counterfactual dual meaning impacts incremental sentence processing.

## 1 Introduction

People often ponder over the alternatives to their earlier decisions and actions, consider unrealized possibilities or engage in mere fabulous imaginations. *What if I had chosen to study another subject? Would Tom have passed the test if he had studied harder? Would I be able to fly if I had wings?* These considerations are examples of counterfactual thought, and the conditional ‘If then’ construction is the canonical form in which such thought is expressed (e.g. Byrne 2002; Roese et al. 2005).

Counterfactual thought is pervasive in everyday life (Roese 1997) and has various adaptive functions. For example, counterfactual thought enables people to reason about the cause of an event (Egan and Byrne 2015; Rips and Edwards 2013; Spellman and Mandel 1999) and thereby plays an important role in the processing of learning from experience (Barbey et al. 2009; Byrne 1997). It also promotes emotions such as regret and relief, and as such helps to regulate behaviour and emotions in order to adequately function in a physical and social environment (Epstude and Roese 2008; Frith 2013). Counterfactual thinking is furthermore associated with the understanding of the perspectives and beliefs of others, which might qualify it as a developmental precursor of explicit Theory of Mind abilities (Peterson and Bowler 2000; Riggs et al. 1998 but see Perner et al. 2004 for an alternative perspective). Counterfactual thought is thus considered to be a highly complex cognitive capability that develops relatively late in childhood (Rafetseder and Perner 2012, 2014) and that is often impaired along with other cognitive functions in clinical conditions like autism, depression, Parkinson and schizophrenia (Grant et al. 2004; Hooker et al. 2000; McNamara et al. 2003; Quelhas et al. 2008).

According to cognitive accounts of counterfactual thought (Fauconnier 1994; Johnson‐Laird and Byrne 2002), the reason that counterfactuals are cognitively complex is that they trigger two incompatible representations. For instance, ‘If I had wings, then I would be able to fly’ expresses (1) the suppositional but factually false state of the speaker having wings and being able to fly, while also expressing (2) that the speaker does not have wings and therefore relies on conventional modes of transportation. This dual meaning is the characteristic feature of counterfactuals. From a linguistic perspective, this dual meaning makes counterfactuality a fascinating phenomenon that enables people to produce utterances that are factually false yet truthful. Counterfactuals hence broaden the scope of communication and allow meaningful conversation about topics beyond mere veridical statements. However, this flexibility in the way people use language may come at a cost. Is the representation of dual meaning costly in terms of maintenance and processing compared to ‘singular’ meaning?

Here, we address this question in relation to language comprehension. How counterfactual dual meaning plays out during language processing is currently gaining interest in psycholinguistics. This may be the case because counterfactual language still poses an important challenge to current models of discourse processing, given that these models do not readily explain the ability to entertain competing representations for extended periods of time (e.g. Kintsch 1988; McKoon and Ratcliff 1998). In particular, counterfactuals comprise several features whose impact on online processing is still debated, including (implicit) negation, non‐factual supposition and (pragmatic) inference generation. Thus, the impact of incompatible factual and counterfactual representations on incremental sentence comprehension remains unclear. What are the language processing correlates of counterfactual dual meaning assumed in cognitive theory? Does dual meaning make a counterfactual more difficult to understand? The present article reviews research that has tackled these questions using online measures (self‐paced reading, eye‐tracking, electroencephalography (EEG) and functional resonance imaging (fMRI)). Online studies focus on the naturally unfolding, incremental extraction of meaning from linguistic information during comprehension. In contrast, offline tasks collect meta‐linguistic judgments after linguistic content has been presented and processed, which makes them particularly useful to study explicit counterfactual reasoning and inference generation. Although such studies repeatedly support the accessibility of counterfactual dual meaning, their results can be distorted by response strategies and meta‐linguistic knowledge. In particular, collecting explicit behavioural responses may introduce processing steps that would not occur during reading for a more ‘regular’ or ‘passive’ mode of comprehension (Chwilla et al. 1995; Hahne and Friederici 2002). The current review therefore focuses on the available evidence for or against dual representation during online counterfactual processing.

### 1.1 What are Counterfactuals?

As the name suggests, counterfactuals are sentences that describe events or situations that are *counter‐to‐fact*, hence factually false. The canonical form to express a counterfactual is a counterfactual conditional, which has a factually false antecedent (i.e. ‘if’ part) that is taken as suppositionally true (Byrne 2002). The antecedent ‘If I had wings’ expresses a non‐factual affirmative state that *I have wings* but also implicitly conveys its negation that *I do not have wings*. In simple (non‐conditional) counterfactuals, this feature can be described as the reversal of the polarity of the initial sentence structure (Van Linden and Verstraete 2008). Similar to conditionals, simple counterfactuals convey a positive statement together with its negation. The utterance ‘I should have called my mother’ conveys the proposition of *me having called my mother* together with a reversed proposition of *me* (in fact) *not having called my mother*. Antecedent falsity is therefore a special case of polarity reversal. Both have the effect that the compositional suppositional proposition becomes enriched by its negation, adding up to a dual meaning.

### 1.2 Pragmatic Accounts of Counterfactual Meaning

Different accounts exist about the linguistic‐pragmatic mechanisms for generating counterfactual dual meaning. For instance, Van Linden and Verstraete (2008) describe polarity reversal of simple counterfactuals in terms of scalar implicatures associated with past‐tense modal expressions (e.g. *would*, *might, should, could*) (see also Ziegeler 2003). Based on the Gricean maxim of Quantity (*Make your contribution as informative as required*; Grice 1975), modal expressions implicate the non‐applicability of the non‐modal, much in the same way that the scalar quantifier *some* pragmatically implicates *not all* (Levinson 2000). On a scalar ordering of informativeness, non‐modal assertions are more informative than modal expressions because non‐modals convey facts rather than mere possibilities. Thus, uttering the informatively weaker modal expression implicates that the epistemically stronger non‐modal does not hold, thereby giving rise to the negated factual meaning.

Other common explanations focus more strongly on counterfactual conditionals and explain antecedent falsity in terms of presuppositions associated with subjunctive mood (Karawani 2014; Levinson 1983; Stalnaker 1975; von Fintel 2012)^1^. Subjunctive mood is a verb form strongly associated (and sometimes equated) with counterfactuality. In the study of conditionals, subjunctive mood (*If Tom had studied hard, he would have passed the test*) historically has been contrasted with indicative mood (*If Tom studied hard, he passed the test*), which expresses hypothetical thought and lacks reference to factual events (Adams 1970). The presupposition account posits that speakers use subjunctive mood to express something that both speaker and listener know to be false. This overt signal prevents the listener to take the false utterance either as a lie (because the speaker does not expect the listener to believe it) or as an error (because the listener knows that the speaker knows that it is false), thereby maintaining the speaker's truthfulness. In cases where the presupposed factual information is initially unknown to the listener, it can be derived from the subjunctive antecedent and accommodated, thus added to the knowledge of the state of affairs.

Linguistic explanations of counterfactual meaning thus assume that some form of pragmatic reasoning, based on cooperative principles between speaker and hearer, is involved in counterfactual sentence comprehension. This harbours possible predictions about the online processing consequences of counterfactuals. Some accounts assume that pragmatic implication generation is costly and only occurs when it is essential for comprehension (Sperber and Wilson 1986). This suggests that not all participants will infer counterfactual dual meaning in all situations. The availability of implied factual meaning would be determined by participants' standard of relevance, which would in turn depend on their cognitive capacities, their sensitivity to social‐communicative cues as well as task‐dependent incentives to derive the inference (Nieuwland et al. 2010). Other accounts assume automatic inference generation but predict increased processing costs once implications have to be cancelled (Levinson 2000). This suggests that increased processing demands should only occur when implied factual meaning is explicitly violated and thus has to be revised. Although the processing consequences implied by pragmatic theories deserve more attention and elaboration in the psycholinguistic study of counterfactuals, they might not fully capture counterfactual dual meaning. This is because they are mainly concerned with the derivation of counterfactuals' ‘second’ (i.e. factual) meaning, while saying little about the suppositional ‘first’ one. Psycholinguistic research has therefore focused on potential processing correlates of counterfactual comprehension as implicitly assumed in cognitive theories.

### 1.3 Cognitive Theory and the Dual Meaning of Counterfactuals

#### 1.3.1 The Mental Space Framework

Fauconnier (1994) formalized the dual representation of counterfactuals within the cognitive semantic framework of mental spaces. Mental spaces are abstract cognitive domains that are used to describe mental representations and processes that underlie thought and language. Within this framework, counterfactual conditionals are considered as mental space builders, carrying information that deviates from their preceding ‘parent’ space representing factual events. Counterfactual mental spaces per definition contain information that is incompatible with their factual parent space, which is also the case for mentalist expressions like false beliefs, unrealized wishes and even negations. The mental spaces framework thus explicitly posits a dual cognitive representation of counterfactuals. However, this framework does not formulate clear, testable predictions regarding dual representation. It does not specify whether and when building of a new mental space incurs a processing cost, or for how long such novel spaces are maintained. Nevertheless, the framework offers an intuitively plausible formalization of counterfactuality and captures the phenomenon of dual meaning in counterfactual conditionals and non‐conditional constructions (e.g. wishes) alike.

#### 1.3.2 Mental Model Theory

Mental model theory (MMT) is a theory of human reasoning put forward by Johnson‐Laird and Byrne (2002) that offers some explicit processing predictions of counterfactual dual meaning. MMT represents the content of reasoning and inference processes as iconic mental representations (so‐called ‘mental models’). Indicative and subjunctive‐counterfactual conditionals trigger different mental models. MMT assumes that people understand an indicative conditional sentence (*If A then B*) by constructing a mental model of the suppositional state only (*A* and *B*). In contrast, people understand counterfactual conditionals by representing two possibilities (Byrne 2005), the suppositional state (*A* and *B*) as well as the implied factual event (*non‐A* and *non‐B*). Reasoning research has indeed revealed judgment differences as a function of the linguistic mood in which conditional premises are presented. Compared to indicative conditionals, subjunctive conditionals more often elicit inferences about the factual model (*non‐A* and *non‐B*), suggesting that factual events are more accessible after counterfactuals.

MMT has not remained unchallenged despite having received a large amount of experimental support (e.g. Byrne and Egan 2004; Byrne and Tasso 1999; Thompson and Byrne 2002). Its major antagonist is the suppositional theory (ST) developed by Evans and colleagues (Evans 2007; Evans and Over 2004). In contrast to MMT, ST denies a general difference between the way indicative and subjunctive conditionals are processed during reasoning. Instead, both kinds of conditionals are assumed to be evaluated with respect to suppositional possibilities in which the consequent (*B*) is true in the context of the antecedent (*A*). As both MMT and ST are theories of conditional reasoning (i.e. inferences carried out with counterfactual premises), they are limited in their application to the study of online sentence comprehension. Although MMT suggests that constructing and manipulating two models require more working‐memory resources compared to one model (Johnson‐Laird and Byrne 1991), it does little to explain how the counterfactual dual model is established in the first place. ST acknowledges that counterfactuals can imply opposite facts, but distinguishes this second meaning as a pragmatic rather than logical aspect which is beyond the scope of the theory (Evans et al. 2005). Whereas both approaches use linguistic content for studying subsequent reasoning behaviour, the psycholinguistics of counterfactual meaning asks how people compute meaning from linguistic content, as online reasoning processes and sentence comprehension unfold hand‐in‐hand.

### 1.4 Offline Studies of Counterfactual Dual Meaning

Offline measures of sentence comprehension consistently suggest that the dual meaning of counterfactuals is accessible after people read counterfactuals. Carpenter (1973) tested how information is extracted from counterfactuals using a sentence‐probe task. Participants read two‐clause sentences in either indicative or subjunctive mood (*Mary stayed, since Judy (would have) lived*) followed by sentence verification probes that either restated or contrasted one of the clauses (*Judy lived* or *Judy died*, respectively). Participants had to indicate whether the probes matched the factual events implied by the sentence. When the probe was presented immediately after the two‐clause sentence, responses to matching probes took longer than responses to mismatching probes following subjunctive but not following indicative sentences. However, when the probe appeared after 5 seconds, all the matching probes triggered faster responses. Carpenter therefore proposed a multistage processing model wherein people initially represent counterfactuals in a negated form (false (*Judy lived*)) and subsequently convert them into an affirmative representation of their factual meaning (*Judy died*). However, the task encouraged participants to systematically ignore counterfactual's suppositional meaning, which made it rather artificial and prone to invite response strategies. Furthermore, the question remains how the implication of a counterfactual without a clear antonym (e.g. *Judy would have laughed*) can be represented more economically, i.e. without negation.

With a sentence recognition task, Fillenbaum (1974) demonstrated that participants infer counterfactually implied factual information even without the explicit instruction to do so. Negative declarative sentences (*He didn*'*t catch the plane*) were incorrectly rated as previously presented more often when they followed a causal counterfactual (*If he had caught the plane he would have arrived on time*) than when they were in fact new. Although this study lacked a matched indicative control condition, this result supports a dual representation view, as the counterfactually implied factual meaning was accessed and memorized before subjects were instructed about the subsequent task and could opt for a response strategy.

A series of studies conducted by de Vega et al. (2012; 2007) addressed how counterfactual dual meaning was maintained over time. Participants read vignettes describing a situation (*John was still in the office sitting in front of the computer. He started to type a report that his boss had asked him for*), followed by a factual (*As he had enough time, he went to the café to drink a beer*) or a counterfactual continuation (*If he had had enough time, he would have gone to the café to drink a beer*). Then a probe was presented after the continuation that was either related to the initial (‘type’) or the new (‘drink’) situation. Positive responses for matching initial‐related probes were faster in counterfactual compared to factual contexts, whereas new‐situation probes did not differ (Experiment 2). Based on these results, de Vega et al. (2007) proposed that counterfactuals momentarily activated a dual representation, which, due to being cognitively demanding, was given up in favour of the representation of initial factual events. The authors argued that the suppositional meaning of counterfactuals therefore did not contribute to the build‐up of a discourse representation. A related study (de Vega and Urrutia 2012) varied the temporal interval between probe‐presentation. A delay of 1500 ms but not of 500 ms produced the initial effect, suggesting that counterfactuals' suppositional meaning was accessible after 500 ms, but not after 1500 ms anymore. The results support the authors' hypothesis that counterfactual meaning is only briefly represented. However, in the employed stimuli, the probe task was always followed by factual sentences, hence the counterfactual reverie was never continued. It may be a strategic adaption to these stimuli that the suppositional meaning was not represented for a longer time, since it was never required.

A more explicit approach to show accessibility to counterfactual dual meaning was used by Thompson and Byrne (2002). Employing a multiple‐choice task which presented various interpretations for each item, the authors asked participants what information was implied by counterfactual conditionals (*If Sarah had gone to Moose Jaw then Tom would have gone to Medicine Hat*). Implication of the opposite factual events was indicated by roughly 50% of participants. This number could be increased to 67% by using causal (*If the butter had been heated, then it would have melted*) and definitional (*If the card had had a jack on it, then it would have been a face card*) content (Experiment 2). The results demonstrate a rather heterogeneous sample, suggesting strong individual differences in the way people assess counterfactual implications. Similarly, Grant et al. (2012) showed that simple declarative sentences with modal verbs also carry a counterfactual meaning. Test sentences (e.g., *This information should be released*) made 89% of participants infer that in fact *The information was not released*, suggesting a counterfactual reading of the sentence. Similar results were obtained for other modals.

Taken together, offline results seem to support the dual representation view. They consistently show that when given the task to access or evaluate counterfactually implied facts, participants tend to do so. However, given the limitations of offline studies, it remains unclear whether dual meaning is computed spontaneously during counterfactual sentence processing or established in order to complete a given task.

## 2 Online Studies of Counterfactual Sentence Processing

In contrast to offline studies, online methods can track counterfactual dual meaning computation using more direct markers of semantic processing on a word‐by‐word basis. Most commonly, counterfactuals have been studied with self‐paced reading, eye‐tracking, EEG and fMRI. In self‐paced reading studies, sentences are presented word‐by‐word or in multi‐word segments, while participants pace the presentation via button press. This aims to measure comprehension by looking at the time participants require to move to the next segment or word. Novel, unexpected or contextually implausible words take longer to process, as also suggested by eye‐tracking studies (Rayner 1998; Rayner 2009). Compared to self‐paced reading, eye‐tracking does not interfere with the natural reading mode by artificially partitioning the stimulus. Instead, participants read a complete sentence or paragraph while their eye‐movements are continuously recorded. This provides reading times per part of the sentences and allows the calculation of additional measures. For instance, participants might re‐read earlier passages to resolve initial difficulties with an encountered word. While self‐paced reading and eye‐tracking measures provide valuable behavioural information, EEG has the great advantage to provide the neurophysiological correlates of language processing by measuring scalp‐recorded electric potentials elicited by neuronal activity. In EEG studies on written language comprehension, sentences are typically presented in a pre‐set pace word‐by‐word. Averaging electric signals that co‐occur with the presentation of a critical word produces event‐related‐potentials (ERPs) with specific latencies, peak amplitudes and topographic distributions. The ERP component most strongly associated with language processing, the N400 (Kutas and Hillyard 1980), peaks around 400 ms after word‐onset and has a centro‐parietal distribution. N400 amplitude is thought to index the ease of retrieving conceptual knowledge associated with a word in a given context (Kutas and Federmeier 2000). Lower (less negative‐going) N400 amplitudes reflect the facilitation of semantic retrieval, for example by a related word or a coherent sentence or discourse. The N400 amplitude therefore also reflects whether a given word is predictable given its context (Kutas and Federmeier 2011). Unfortunately, EEG does not allow the precise localization of neural activity that produces the scalp‐recorded ERPs. Therefore, fMRI has been employed in the study of sentence processing, even though its temporal imprecision does not qualify it as an online measure. With the temporal resolution of about 2 seconds fMRI provides spatially fine‐grained information about brain regions that are involved in language processing. All these online measures are collected while participants are reading (or listening to) a narrative with no principled need of an additional task. The accessibility of a concept can therefore be measured at each word during the incremental build‐up of sentence meaning without potential interference from task‐oriented processing.

The dual representation of factual and suppositional meaning during counterfactual sentence comprehension could have various processing consequences. On one hand, the initial build‐up of dual meaning could be costly compared to ‘singular’ meaning, because dual meaning requires a more elaborate situation model that encompasses the representation of explicitly contradicting information. These increased processing demands might occur early during counterfactual sentence processing, when people process the counterfactual antecedent. On the other hand, once counterfactual dual meaning is computed, it would increase the accessibility and facilitate the processing of information that is related to both meanings. Online studies of counterfactual sentence comprehension can thus be roughly grouped by whether the words at which processing is probed (i.e. critical words) are located in the counterfactual antecedent where dual meaning is constructed, or in the counterfactual consequent or even in a subsequent sentence.

### 2.1 Processing the Counterfactual Antecedent

Only a few studies have investigated the accessibility of a counterfactual meaning by collecting online measures during the processing of counterfactual antecedents. In a self‐paced reading‐time study, Stewart et al. (2009; see also Haigh and Stewart 2011) presented three different types of context sentences (*Darren was not athletic/was very athletic/enjoyed meeting new people*), which rendered a subsequent subjunctive conditional (*If Darren had been athletic, he could have tried out for the rugby team*) contextually consistent, inconsistent or neutral. The critical segment within the antecedent (*athletic, he*) was read faster when it was contextually consistent than when it was inconsistent or neutral. This result suggests that counterfactual antecedents are rapidly evaluated with respect to previous information, with mismatch between counterfactually implied and explicitly stated factual events resulting in slower reading. No such slow‐down was observed for contextually inconsistent indicative conditionals (*If Darren was athletic, he tried out for the rugby team*), suggesting that readers relate indicative and subjunctive conditionals to contextual information in different ways. In particular, readers expect the subjunctive antecedent to be false in respect to prior information, suggesting an immediate effect of counterfactuals' pragmatic constrains of antecedent falsity on the unfolding discourse. Interestingly, readers also read the neutral antecedent slower than the contextually consistent antecedent. This effect was taken to reflect implication generation in order to infer factually implied events of the counterfactual antecedent. However, no neutral control condition in indicative mood was presented to dissociate whether increased reading times were in fact due to counterfactual dual meaning. Therefore, the results may only reflect the lack of lexical overlap and discourse coherence between the neutral context sentence and the counterfactual antecedent.

In an ERP study on German sentence processing, Kulakova et al. (2014) investigated the processing of counterfactual and indicative antecedents without preceding context. The authors examined whether counterfactual antecedents are associated with additional processing costs due to dual meaning computation. They compared ERPs to verbs in subjunctive/indicative mood (*Wenn die Würfel gezinkt wären/waren*; translation: *If the dice had been rigged/were rigged*). The subjunctive mood verbs (*wären*) elicited a left‐frontal negative deflection in the 450–600 ms time window compared to the indicate mood verbs. The authors interpreted this effect as an instance of the left anterior negativity (LAN), a physiological signature of increased working memory demands in syntactic but also semantic processing (Krott and Lebib 2013; Matzke et al. 2002). Under this interpretation, the incremental computation of counterfactual antecedents increases working memory demands as soon as counterfactuality becomes apparent. Whereas the observed ERP effect could not be explained by cloze or frequency values of the critical verbs, further research is needed to tease apart the effects of linguistic mood from effects of the specific word form. In addition, the nature of the cognitive processing costs (inference generation, inhibition of factual knowledge, dual meaning maintenance, etc.) remains to be identified.

Taken together, there is indirect support of increased processing costs associated with dual meaning generation in counterfactual antecedents. However, none of the available studies completely rule out alternative explanations for these effects.

### 2.2 Processing Narrative Following a Counterfactual Context

The majority of studies on online counterfactual sentence comprehension investigated the impact of a counterfactual context on the processing of subsequent phrases. For example, Santamaría et al. (2005) (see also Gómez‐Veiga et al. 2010) presented either indicative or counterfactual context sentences (*If there are/had been roses, then there are/had been lilies*) and measured participants' (self‐paced) reading times for subsequent affirmative or negated declarative sentences about these events (e.g. *There were (no) roses, and there were (no) lilies)*. Participants read negated sentences faster after subjunctive compared to indicative conditionals, whereas no difference occurred for affirmative sentences. These results can be taken to reflect dual meaning, as counterfactuals facilitated the reading of the implied (negated) factual events without slowing down the processing of supposed (positive) events.

In a related eye‐tracking experiment, Ferguson (2012) presented counterfactual conditional or factual declarative context sentences (*If/Because Joanne had remembered her umbrella, she would have had/had avoided the rain*) followed by a factual sentence that included a critical word that was either consistent or inconsistent with the preceding context (*Joanne's hair was dry/wet*). One early measure of processing difficulty showed that counterfactual inconsistencies were immediately detected, increasing the tendency to re‐read earlier regions. However, the general pattern showed that critical words were read fastest when they were consistent with the factual context and slowest when they were inconsistent with the factual information. In contrast, following a counterfactual context, both consistent and inconsistent critical words were read equally slow, but faster than inconsistent words in a factual context. Contextual consistency with a factual context thus had different effects than consistency with a counterfactual context, which the author attributed to a dual representation of counterfactuals. A sustained representation of dual meaning was assumed to make counterfactuals more demanding in terms of processing resources, leaving fewer resources to anomaly detection. These results are intriguing, although alternative explanations are possible. It remains to be examined whether these findings reflect difficulty with processing conditionals (compared to factual sentences) or whether they are indeed specifically reflecting counterfactual meaning. It is also possible that participants had difficulty switching from a counterfactual scenario back to factual events.

Similar findings were presented in an EEG study that used materials similar to those from de Vega's (2007) offline design (Urrutia et al. 2012a). An initial situation (*Marta wanted to plant flowers*) was followed by either a factual (*Because she found a spade, she started to dig a hole*) or a counterfactual (*If she had found a spade, she would have started to dig a hole*) continuation. EEG was recorded over multi‐word intervals of subsequent phrases that were either consistent with initial (*Marta bought a spade in the market*) or new events (*Marta planted some roses in the ground*). Initial‐related continuations elicited increased frontal negativities after (inconsistent) factual compared to (consistent) counterfactual contexts. In contrast, counterfactual‐inconsistent (new‐related) phrases did not show such effect. Unfortunately, initial and new continuations were not matched for lexical‐semantic factors, preventing a clear interpretation of this finding. Furthermore, the frontal distribution of the sustained negativity suggests an effect other than an N400 effect. The authors argued for a similarity with frontal effects elicited by ambiguous words that are held in working memory until the point of disambiguation (Hagoort and Brown 1994), which is reminiscent of the working memory‐related negativity‐effects described by Kulakova et al. (2014) in counterfactual antecedents. Taken together, the studies of Ferguson (2012) and Urrutia et al. (2012a) point at increased working memory demands elicited by counterfactual sentences.

In a recent ERP study, Ferguson and Cane (2015) directly investigated the impact of individual working memory capacity on the processing of narrative following counterfactuals. Participants read counterfactual or factual sentences (*If/Because David had been wearing his glasses, he would have read/was able to read the poster easily*) followed by simple factual or counterfactual phrases (*From this distance, David* (*would have*) *found that the words were clear/blurry*) manipulating consistency. The authors investigated N400 effects of contextual consistency in three combinations of the two sentences: factual‐factual, counterfactual‐counterfactual and counterfactual‐factual. The low working‐memory group showed N400 inconsistency effects only in factual‐factual conditions. In contrast, high working‐memory participants showed inconsistency effects in both factual‐factual and counterfactual‐counterfactual conditions. Although no online effect of inconsistency was observed in counterfactual‐factual conditions, offline cloze ratings indicated that participants produced consistent continuations when given enough time. The authors concluded that representing counterfactual narrative was cognitively more demanding compared to processing factual sentences, so that only high working‐memory participants showed inconsistency effects after a counterfactual context. Switching from counterfactual to factual phrases was argued to be even more effortful and as such no inconsistency effects occurred even in the high working‐memory group. These results substantiate the interpretation of earlier findings (Ferguson 2012). Further studies are required to establish whether these findings reflect the impact of working memory capacity on counterfactual dual meaning or rather on more general processes such as attention or depth of processing.

The counterfactual materials discussed so far all describe realistic situations, alternative courses of events that could realistically have taken place. However, some counterfactuals reflect considerations of impossible events (e.g. *If I had wings)*. For such sentences, the factual meaning (*I don't have wings*) is directly accessible from general real‐world knowledge. Several studies investigated how real‐world knowledge influences the build‐up of counterfactual meaning, in particular how people evaluate the consequences of counterfactuals that contradict factual world knowledge. In an eye‐tracking study, Ferguson and Sanford (2008; Experiment 2) presented counterfactual context sentences that were inconsistent with world knowledge (*If cats were vegetarians they would be cheaper for owners to look after*). These were either followed by a sentence that was contextually consistent but contradicted world knowledge (*Families could feed their cat a bowl of carrots*), or a sentence that was inconsistent with the counterfactual context but consistent with world knowledge *(feed their cat a bowl of fish*). The control condition was a factual context sentence (*Evolution dictates that cats are carnivores and cows are vegetarians*). Similar to the results of Ferguson (2012), the world knowledge consistency effect was greatest following the factual context, whereas total reading times did not reveal an effect of counterfactual consistency. These results suggest that representation of counterfactual dual meaning is cognitively demanding and reduces anomaly detection. However, a replication with slightly different stimuli (*could* was replaced by *would*; Experiment 3) yielded different results. Following counterfactual context, consistent words were read longer than inconsistent words, whereas this pattern was reversed following factual context. This result suggests that real‐world knowledge was more accessible than the consequences consistent with the counterfactual situation. In an ERP study, Ferguson et al. (2008) probed the same critical words (*carrots* vs. *fish*) following a counterfactual context. The consistent word *carrots* triggered stronger N400 amplitudes. Unfortunately, the critical words were not controlled for predictability and semantic relatedness with the preceding context, both variables known to influence the N400 amplitude (Federmeier et al. 2007). This makes it difficult to decide whether in both the eye‐tracking and ERP studies the word *carrots* was more difficult to process because it was false in respect to world knowledge or simply because another counterfactual‐consistent word (e.g. *vegetables*) was expected instead (for discussion, see Nieuwland and Martin 2012). In fact, an eye‐tracking study in which similar, spoken sentences were accompanied by pictures of carrots or fish (but no other edible items) showed that participants looked at the consistent stimulus of both counterfactual and factual conditions right after the onset of the verb (*feed*). This speaks for an early contextual expectation effect without any bias towards world knowledge (Ferguson et al. 2010).

Nieuwland and Martin (2012) examined a related question in an EEG study using critical words that were equally expected from a counterfactual or a real‐world context. Participants read counterfactual or factual sentences regarding historical events (*If N.A.S.A. had not developed its Apollo Project, the first country to land on the moon would have been Russia/America, surely/Because N.A.S.A. developed its Apollo Project, the first country to land on the moon was America/Russia, surely*). The critical word in the consequent (*Russia/America*) was either consistent or inconsistent with the initial scenario. Although counterfactually inconsistent words referred to factually true events, they produced significantly higher N400 amplitudes than counterfactually consistent but factually true critical words. This suggests that the counterfactual context completely eliminated the difficulty to integrate a factually false consequent. Nieuwland (2013) extended this finding to biologically and physically absurd counterfactuals (*If dogs had gills, Dobermans would breathe under water without problems*). ERPs elicited at the critical word (*water*) did not differ from factually true sentences (*Because fish have gills, tuna breathe under water without problems*). In general, these results seem to be at odds with a dual representation of counterfactuals, as only the suppositional meaning influenced critical word processing. However, these studies used different control conditions in order to establish the N400 effect between consistent and inconsistent words, either the factually true alternative (*America*) or a semantically unrelated (*poison*) word. It is possible that although the counterfactually consistent word (*Russia)* is processed more fluently than the factually true word (*America)*, an unrelated word (e.g. *Austria*) is even more difficult to process. Likewise, *Dobermans would breathe under air* may be counterfactually incongruent and unexpected, but still more accessible than *poison*. This makes it difficult to relate these results to counterfactual dual meaning, as they were designed for a different purpose and lack a critical contrast.

### 2.3 Functional Neuroimaging of Counterfactual Sentence Comprehension

Functional neuroimaging studies show that counterfactual thinking and reasoning rely on multiple cognitive functions including mentalizing, cognitive control and emotional processing (see Van Hoeck et al. 2015 for review). The majority of fMRI studies used short cues that referred to (autobiographical) situations to trigger counterfactual evaluation and reasoning processes (De Brigard et al. 2015; Van Hoeck et al. 2010, 2012). In contrast, only few studies explicitly investigated counterfactual sentence processing and can speak to the present question of counterfactual dual meaning.

Nieuwland (2012) employed the historical counterfactual stimuli from Nieuwland and Martin (2012) in an fMRI study. Counterfactuals activated brain regions in bilateral middle temporal gyri to a stronger extent than factual sentences. These regions are involved in the retrieval of word meaning from long‐term memory and sentence‐level integration (Snijders et al. 2010; Price 2010). More focused analyses in frontal regions revealed differential responses to consistency violations in counterfactual compared to factual sentences. Inconsistent counterfactual sentences elicited stronger frontal activations that were more pronounced in the right compared to the left hemisphere. These effects were not detectable with EEG (Nieuwland and Martin 2012), which highlights the advantage of employing different methods that are sensitive to the activation of different neural populations at varying time‐scales. The results speak for the specific involvement of the right hemisphere during counterfactual sentence comprehension. The right hemisphere is assumed to contribute to inferencing and establishing contextual coherence during sentence processing (Jung‐Beeman 2005; Kuperberg et al. 2006; Virtue et al. 2006). In turn, right‐hemisphere damage is associated with pragmatic language deficits (Martin and McDonald 2003).

The prevalent involvement of the right hemisphere was further supported by an fMRI study by Kulakova et al. (2013). Factual sentences (*The motor is switched off today*) were followed by either a counterfactual conditional that explicitly contradicted the presented facts (*If the motor had been switched on today, would it have burned fuel?*) or by a conditional in indicative mood that did not refer to the initial events (*If the motor was switched on yesterday, did it burn fuel?*). In contrast to indicative sentences, counterfactuals activated right lateralized regions in the cuneus and caudate nucleus. As stimuli were presented in two modalities – aurally and visually – the activation in occipital regions could not be attributed to visual perception. Instead, it was assumed to mirror mental imagery processes. These findings were taken to support a dual representation of counterfactuals as proposed by MMT, with increased iconic representation demands of the counterfactual sentence.

Taking an embodied perspective, Urrutia et al. (2012b) tested the difference between the sensory‐motor representation of counterfactuals and factual sentences. Participants listened to factual and counterfactual statements referring to actions that varied in their degree of motor effort (*Since/If Pedro* (*had*) *decided to paint the room, he is moving/would have moved the photograph/sofa*). Regardless of physical effort, counterfactuals elicited increased activations in left superior frontal regions, bilateral hippocampal gyri, as well as right inferior temporal gyrus. The authors concluded that more cognitive effort was required to process counterfactual compared to factual sentences. Since two meanings of counterfactuals were assumed to be active at the same time, frontal activations were interpreted to reflect the cognitive cost of managing or inhibiting two conflicting action representations. In this comparison, however, effects of different sentence structure cannot be disentangled from supposed effects of counterfactual dual meaning.

So far, fMRI studies of counterfactual sentence processing show a rather fragmented picture. Nevertheless, one emerging pattern is that counterfactuals elicit stronger brain activations compared to their factual or hypothetical control conditions. In fact, none of the reported studies found regions that were activated stronger by the opposite contrasts. This is in line with a more costly representation or higher processing demands of counterfactuals. However, more studies are required to identify the exact cognitive cause of the surplus activation. A more focused prediction could be that if counterfactual and factual representations are in competition with one another, counterfactuals should elicit increased activations in brain regions associated with semantic competition and semantic selection such as the left inferior prefrontal cortex (e.g. Thompson‐Schill et al. 2005).

## 3 Conclusion

We started this review with an overview of the dual meaning assumption in cognitive and linguistic accounts of counterfactual meaning. In fact, there seems to be so little doubt about a dual meaning in theory that it is rather regarded as a defining characteristic of counterfactuals than a property to be demonstrated experimentally. The experimental question regarding online sentence processing is therefore not whether counterfactuals can convey a dual meaning (offline studies established that they can), but whether they always do and how exactly such dual meaning relates to incremental build‐up of sentence meaning. The available online sentence comprehension studies show that counterfactual antecedents require longer processing time when they contain novel factual meaning as opposed to given factual meaning. They also show that counterfactual events are immediately checked for consistency with prior discourse. Furthermore, counterfactual scenarios are quickly incorporated into the representation of the discourse so that they impact the comprehension of successive narrative. This process seems to draw on working memory resources. However, online evidence for a synchronously dual representation of counterfactual meaning at a specific point in time remains indirect.

Because the reviewed experimental findings are diverse and sometimes contradictory, interpretation of the results must rely on the specifics of the counterfactual materials as well as the control materials. Counterfactual sentences are usually contrasted with factual declarative sentences (*Because A, B*) or with indicative conditionals (*If A, then B*). From the viewpoint of incremental sentence processing, indicative conditionals may constitute a better control condition in terms of matching conditional sentence‐structure and suppositional meaning. This is important, because observed differences in online processing can be attributed to dual meaning with more certainty when other differences are kept minimal. Another not often taken but promising approach in the online investigation of counterfactual dual meaning is to focus on counterfactual antecedents (Kulakova et al. 2014; Stewart et al. 2009). In the antecedent, both the compositional meaning and its pragmatic enrichment are conveyed for the first time and can exhibit potential processing consequences. Studying counterfactual antecedents is therefore better suited to capture the potential computation processes associated with dual meaning than investigating counterfactuals' downstream consequences.

A more fundamental problem for research on counterfactual dual meaning during language comprehension is its imprecise definition in cognitive theory and a missing link between cognitive theory and incremental accounts of language comprehension. Although the theories of mental spaces and mental models are probably the main cognitive reference for counterfactual dual meaning computation, they both lack a direct translation into language processing mechanisms and therefore cannot offer clear processing predictions for incremental counterfactual sentence processing. Hence, what would count as direct online evidence for dual meaning from a sentence‐processing perspective? And, even more important, how can the notion of dual meaning be falsified with online data? In general, there is no reason to expect that cognitive categories map on brain physiology or become distinguishable during online language processing (Page 2006). For dual meaning computation to be detectable with current psycholinguistic methods, it has to be computationally different (e.g. costly) compared to singular meaning and to occur at a specific point or interval during sentence build‐up. On the other hand, if online measures fail to identify or support a neural process that can reasonably be associated with dual meaning computation, this would not guarantee the absence of such a computation as it might be variable in its time course or strongly differ between individuals. Studying counterfactual dual meaning with functional imaging is associated with similar problems (Coltheart 2006; Poldrack 2010). Although convergent evidence for increased processing costs can potentially be gained from different methodologies and provide more precise descriptions of the neural implementation of counterfactual (dual) meaning computation, little can be done to convincingly refute dual meaning. This is because the notion of dual meaning has without notice become a defining characteristic of counterfactuality even in psycholinguistic research and this leads to circularity. Researchers design counterfactual stimuli with two meanings and then interpret processing costs associated with counterfactuals as evidence of their dual meaning. It is important to acknowledge this to move forward with meaningful experimentation.

In our view, this observation does not make neurolinguistic research on counterfactual dual meaning futile. Rather it invites further online investigations to focus on the lack of bridging assumptions between counterfactual dual meaning and online sentence processing. Online studies can aim to clarify the how and when of dual meaning computation. This requires the formulations of testable assumptions of how counterfactual dual meaning relates to incremental sentence‐build‐up. Is it necessarily represented in parallel or are factual and suppositional aspects of counterfactually accentuated in succession? What are the individual differences that account for heterogeneous responses in offline studies that ask what information is implied by counterfactuals (e.g. Thompson and Byrne 2002) and do they also have an impact on online processing? Subjects' sensitivity to contextual constraints and other pragmatic abilities (e.g. Ferguson et al. 2014; Nieuwland et al. 2010) as well as working memory capacity (Ferguson and Cane 2015) are possible candidates.

In the light of the emerging refined pragmatic accounts of counterfactuality, further attempts should also be made to relate counterfactuals to more popular pragmatic phenomena like presuppositions or scalar inferences. The link between pragmatic skills and mentalizing (Cummings 2013) could further help to understand why counterfactual thinking and Theory of Mind abilities develop hand‐in‐hand (Peterson and Bowler 2000; Riggs et al. 1998) even beyond the effect of executive functions (Drayton et al. 2011, Müller et al. 2007). One possibility is that pragmatic language draws on basic mentalizing skills (Sperber and Wilson 1986) and in turn influences or predicts the development of explicit perspective‐taking. Similarly, autists' problems with counterfactual reasoning (Grant et al. 2004; Leevers and Harris 2000) might stem from pragmatic deficits that impair successful counterfactual comprehension leading to further problems with reasoning. Another future aim of experimental research on counterfactuals is to establish an empirical link to the processing of simple modals, wishes and specifically negations given that all these constructions convey a form of counterfactuality.

In sum, online studies of counterfactual dual meaning have and will provide valuable information about the way counterfactual meaning unfolds in time and influences successive information processing. Further advances in research on counterfactual comprehension require more specific bridging theories about how counterfactual dual meaning impacts incremental sentence processing.

## Notes

1 Given the ongoing debate about the mechanisms for computing counterfactual meaning, we will neutrally refer to the process of polarity reversal as an ‘implication’, which is not meant as a synonym for linguistic notions like entailment, implicature or presupposition.
